# Editorial: Innovative modeling and simulation in thrombosis and hemostasis: enhancing diagnosis and treatment

**DOI:** 10.3389/fcvm.2026.1958633

**Published:** 2026-08-24

**Authors:** Rodrigo Assar, Daria Hemmerling

**Affiliations:** 1 Instituto de Ciencias Biomédicas, Facultad de Medicina, Universidad de Chile, Santiago, Chile; 2 Faculty of Electrical Engineering, Automatics, Computer Science and Biomedical Engineering, AGH University of Krakow, Krakow, Poland

**Keywords:** thrombosis, hemostasis, clinical decision support, computational modeling, precision medicine, risk stratification, biomarker integration, machine learning

## Abstract

Computational modeling has become an increasingly important component of thrombosis and hemostasis research, evolving from mechanistic simulations into multidisciplinary approaches that support diagnosis, risk stratification, and therapeutic decision-making. The fourteen contributions included in this Research Topic illustrate this evolution through complementary methodologies, including mechanistic modeling, computational fluid dynamics, machine learning, quantitative imaging, biomarker integration, real-world data analysis, and evidence synthesis. Collectively, these studies demonstrate how biological understanding, individualized prediction, comprehensive patient characterization, and rigorous validation represent interconnected steps toward clinically meaningful computational tools. Rather than competing methodologies, these approaches address complementary dimensions of thrombosis and hemostasis, contributing to more integrated frameworks for precision medicine. Together, this collection highlights both the progress achieved and the challenges that remain in translating computational innovation into robust clinical decision-support systems.

## 1 Introduction

Thrombosis and hemostasis arise from intricate interactions among coagulation pathways, platelet activation, vascular biology, blood flow, inflammation, and patient-specific clinical characteristics. Disruption of this tightly regulated system may result in thrombotic or hemorrhagic complications that remain major causes of morbidity and mortality worldwide. Although remarkable progress has been made in elucidating the molecular basis of these disorders, translating this knowledge into individualized diagnosis and treatment remains a major scientific and clinical challenge.

Computational modeling has become one of the principal strategies for bridging this gap. Initially centered on mathematical descriptions of coagulation kinetics and blood flow, the field has progressively expanded to include computational fluid dynamics, statistical learning, machine learning, quantitative imaging, and large-scale analysis of clinical data. Consequently, modeling is no longer limited to simulating biological mechanisms; it increasingly encompasses computational representations of patient risk, disease phenotypes, procedural performance, and therapeutic decision-making.

The fourteen studies included in this Research Topic illustrate this evolution. Despite employing diverse computational methodologies and addressing different clinical questions, they reveal a coherent progression in computational thrombosis research. Collectively, they demonstrate how mechanistic understanding supports individualized prediction, how richer patient characterization strengthens predictive performance, and how rigorous validation ultimately determines clinical usefulness. The following sections discuss these four interconnected themes and their implications for precision medicine.

## 2 Modeling biological and physical mechanisms

Reliable computational prediction begins with biological understanding. Although recent advances in artificial intelligence and machine learning have transformed thrombosis research, mechanistic models remain essential because they explain the biological and physical processes that predictive algorithms ultimately seek to recognize.

Three complementary studies illustrate this concept across distinct biological scales. Liu et al. modeled coagulation dynamics through a refined phenomenological representation of viscoelastic clot formation and fibrinolysis in trauma-induced coagulopathy, demonstrating how biologically interpretable models may support rapid assessment of coagulation abnormalities. At the vascular level, Sun et al. used computational fluid dynamics to show that multidirectional wall shear stress is associated with thrombotic risk in isolated coronary artery ectasia, emphasizing the importance of local hemodynamic environments in thrombus formation. At the procedural level, Ernst et al. introduced automated quantification of thrombus deformation during mechanical thrombectomy, transforming qualitative observations into objective measurements of clot-device interaction.

Together, these studies demonstrate that thrombosis cannot be adequately represented at a single level of analysis. Molecular reactions, blood-flow dynamics, vascular biomechanics, and device interactions continuously influence one another. Importantly, mechanistic models do more than reproduce biological processes—they provide the physiological rationale that makes computational predictions interpretable and clinically credible.

Mechanistic understanding alone, however, does not determine which individual patients will develop thrombosis or bleeding complications. Translating biological knowledge into patient-specific prediction represents the next stage in the evolution of computational thrombosis research.

## 3 From clinical variables to individualized risk estimation

A central translational objective of computational modeling is to support clinical decision-making. Consequently, computational prediction has become a rapidly expanding area of thrombosis research, with increasing emphasis on individualized rather than universal risk assessment.

Across diverse clinical scenarios—including coronary artery disease, gastrointestinal surgery, pediatric critical care, orthopedic trauma, and prophylactic anticoagulation—the studies in this collection consistently demonstrate that thrombotic and bleeding risk are highly context dependent. Yang et al. and Huang et al. applied machine-learning approaches to predict venous thromboembolism in patients hospitalized with coronary artery disease and postoperative deep vein thrombosis after gastrointestinal surgery, respectively. Zhou et al. developed a dedicated prediction model for catheter-related thrombosis in critically ill pediatric patients, whereas Li et al. demonstrated the contribution of inflammatory indices such as the platelet-to-lymphocyte ratio in femoral fracture patients. Complementing thrombotic prediction, Fu et al. addressed the equally important challenge of predicting major gastrointestinal bleeding after prophylactic anticoagulation.

Rather than supporting the development of a single universal prediction model, these studies collectively argue that computational tools should be adapted to the biological mechanisms, patient populations, and therapeutic contexts in which they will ultimately be applied. Risk is increasingly understood as dynamic, disease-specific, and dependent on evolving clinical conditions rather than as a fixed baseline characteristic.

Yet predictive performance depends not only on computational methodology but also on the biological information available for each patient. This raises another fundamental question addressed by this Research Topic: what information should future computational models incorporate?

## 4 Expanding the phenotype through biomarkers and real-world data

Prediction is constrained not only by computational methodology but also by how completely patients are characterized. Regardless of the sophistication of the algorithm employed, models cannot identify information that is absent from the available data.

Several studies demonstrate how richer biological characterization can strengthen computational prediction. Huang et al. combined thromboelastography with conventional coagulation indicators to improve assessment of thromboembolic risk in patients with cancer, illustrating how functional measurements complement traditional laboratory testing. Hung et al. leveraged a large multicenter real-world database to identify iron deficiency anemia as a clinically relevant factor associated with venous thromboembolism, highlighting the value of population-scale clinical data. Chen et al. characterized the heterogeneous etiologies of extreme thrombocytosis and their relationship with coagulation abnormalities, emphasizing that similar laboratory findings may reflect fundamentally different biological mechanisms. At the healthcare-system level, Yang and Qin analyzed patterns of hospital-acquired venous thromboembolism, providing epidemiological insights capable of informing institutional prevention strategies.

These investigations establish an important connection with the prediction studies discussed previously. Improvements in predictive performance arise not only from increasingly sophisticated algorithms but also from richer representations of patient biology. Biomarkers, functional assays, imaging, and longitudinal clinical information expand the computational phenotype available for modeling, enabling more comprehensive characterization of thrombotic disease.

As computational models incorporate increasingly rich patient phenotypes, attention naturally shifts from model development toward implementation. The next challenge is determining whether these increasingly sophisticated approaches can be translated into robust and clinically trustworthy decision-support tools.

This multidimensional characterization of thrombotic disease naturally points toward future integration of multimodal clinical information—including laboratory biomarkers, imaging, longitudinal health records, and potentially genomic or proteomic data—within unified computational frameworks.

## 5 From predictive performance to trustworthy clinical implementation

The transition from promising computational models to routine clinical tools remains a major challenge in precision medicine. High predictive performance alone is insufficient unless models are trustworthy, reproducible, externally validated, and capable of improving patient care.

This perspective is reinforced by the systematic review and meta-analysis by Yang et al., which critically evaluated prediction models for catheter-related thrombosis in cancer patients. Despite encouraging discrimination reported by many studies, the review identified substantial methodological limitations and high risks of bias, reminding us that model development represents only the first step toward clinical translation. Rather than contradicting the original predictive studies included in this Research Topic, this review reframes them by emphasizing the importance of rigorous validation, transparent reporting, and methodological robustness.

A complementary perspective is provided by Mao et al., whose review of perioperative management of direct oral anticoagulants illustrates that even accurate computational predictions cannot replace individualized clinical judgment. Instead, computational approaches should support clinicians in balancing thrombotic and bleeding risks while integrating patient characteristics, procedural factors, and evolving evidence.

Importantly, this final theme connects all preceding sections. Mechanistic models improve biological interpretability; richer patient characterization strengthens predictive information; context-specific prediction increases clinical relevance; and rigorous evaluation determines whether these advances ultimately improve patient care. Clinical implementation therefore represents the convergence of the entire computational modeling pipeline rather than a separate methodological objective.

## 6 Conclusions and future perspectives

Beyond the individual findings presented by each study, this Research Topic reveals a broader transformation in computational thrombosis research. The field is progressively evolving from isolated modeling approaches toward integrated computational ecosystems that connect biological mechanisms, patient phenotypes, clinical prediction, and therapeutic decision-making.

Although the contributions employ markedly different computational methodologies—including mechanistic simulation, computational fluid dynamics, machine learning, statistical modeling, quantitative imaging, biomarker integration, and evidence synthesis—they should not be viewed as competing paradigms. Rather, they address complementary dimensions of thrombosis and hemostasis, collectively contributing to a more comprehensive computational representation of disease biology and clinical decision-making.

Prediction without biological understanding lacks interpretability, whereas biological understanding without prediction has limited clinical applicability. Precision medicine requires both. Future progress will depend not only on algorithmic sophistication, but also on biological grounding, multimodal patient characterization, rigorous validation, and prospective clinical evaluation. Hybrid mechanistic–machine learning models, multimodal data integration, digital twins, explainable artificial intelligence, and continuously updated prediction systems represent particularly promising directions.

Rather than representing independent methodological advances, the studies assembled in this Research Topic describe successive steps toward the same objective: translating computational innovation into clinically meaningful precision medicine. Continued collaboration among clinicians, engineers, computational scientists, statisticians, and biologists will be essential to transform increasingly sophisticated computational models into robust clinical tools that demonstrably improve clinical outcomes, safety, or workflow.

